# Postvaccination COVID-19 among Healthcare Workers, Israel

**DOI:** 10.3201/eid2706.210410

**Published:** 2021-06

**Authors:** Zohaib Yousaf, Kamran Mushtaq

**Affiliations:** Hamad Medical Corporation, Doha, Qatar; Dresden International University, Dresden, Germany

**Keywords:** coronavirus disease, COVID-19, severe acute respiratory syndrome coronavirus 2, SARS-CoV-2, coronaviruses, viruses, respiratory infections, vaccination, postvaccination, vaccines, diagnosis, healthcare workers, zoonoses, Israel

**To the Editor:** We read with interest the article by Amit et al. (*1*), in which the authors discuss postvaccination symptoms as harbinger for severe acute respiratory syndrome coronavirus 2 (SARS-CoV-2) infection in healthcare workers (HCWs). The authors conclude that in HCWs, SARS-CoV-2infection can overlap with postvaccination symptoms caused by high risk for exposure. We agree with the authors regarding the dilemma posed by physical symptoms after vaccination. However, we emphasize that this situation extends beyond HCWs.

We report a 60-year-old previously healthy man who had isolated, progressively worsening fatigue starting 1 day after receiving a second dose of mRNA-based vaccine (Pfizer/BioNTech, https://www.pfizer.com). Reverse transcription PCR of a nasopharyngeal swab sample after fatigue persisted beyond 1 week of vaccination showed a positive result for SARS-CoV-2. The patient later showed development of severe coronavirus disease (COVID-19) pneumonia, requiring admission to an intensive care unit.

HCWs are at a higher risk for exposure and more likely to get tested if symptoms develop after vaccination, as in the study by Amit et al. (*1*). Fatigue is a common symptom after receiving the Pfizer/BioNTech vaccine (*2*). The dilemma associated with persistent postvaccination symptoms is magnified in non-HCWs because of perceived low overall exposure risk. A watch-and-wait strategy with false reassurances caused by postvaccination status in these patients might delay PCR testing, leading to further spread of the virus in the community (D.A. Swan et al., Fred Hutchinson Cancer Research Center, pers. comm., 2021 Mar 5). Thus, knowing when and whom to test postvaccination has major public health repercussions.

COVID-19 vaccines are efficacious in reducing transmission of symptomatic COVID-19 (*2*). Reduction in transmissibility of SARS-CoV-2 postvaccination is unclear (*3*). Vaccinated persons can still transmit the disease even if they are asymptomatic. (*4*). Testing everyone before and after vaccination for SARS-CoV-2 is not a cost-effective strategy. Being socially responsible is the key to contain the pandemic. Thus, preventive measures such as hand hygiene, use of facemasks, and avoiding social gatherings should be continued irrespective of vaccination status.

## References

[1] Amit S, Beni SA, Biber A, Grinberg A, Leshem E, Regev-Yochay G. Postvaccination COVID-19 among healthcare workers, Israel. Emerg Infect Dis. 2021;27:1220–2. 10.3201/eid2704.210016 33522478 PMC8007324

[2] Polack FP, Thomas SJ, Kitchin N, Absalon J, Gurtman A, Lockhart S, et al.; C4591001 Clinical Trial Group. C4591001 Clinical Trial Group. Safety and efficacy of the BNT162b2 mRNA COVID-19 vaccine. N Engl J Med. 2020;383:2603–15. 10.1056/NEJMoa2034577 33301246 PMC7745181

[3] He X, Lau EHY, Wu P, Deng X, Wang J, Hao X, et al. Temporal dynamics in viral shedding and transmissibility of COVID-19. Nat Med. 2020;26:672–5. 10.1038/s41591-020-0869-5 32296168

[4] Bleier BS, Ramanathan M Jr, Lane AP. COVID-19 vaccines may not prevent nasal SARS-CoV-2 infection and asymptomatic transmission. Otolaryngol Head Neck Surg. 2021;164:305–7. 10.1177/0194599820982633 33320052

